# Retinal Vessel Local Tortuosity under a Macula-to-Optic Disc Central-Framing Change

**DOI:** 10.3390/diagnostics13061030

**Published:** 2023-03-08

**Authors:** Natalia Ramírez, Miquel Ralló, Maria S. Millan

**Affiliations:** Applied Optics and Image Processing Group (GOAPI), Universitat Politècnica de Catalunya-BarcelonaTech, ViolinistaVellsolà 37, 08222 Terrassa, Barcelona, Spain

**Keywords:** tortuosity index, retinography, vessel segmentation, digital eye fundus image

## Abstract

Some ocular and cardiovascular diseases can be detected through the increased tortuosity of retinal blood vessels. Objective tortuosity measures can be obtained from digital image analysis of a retinography. This study tested a set of local tortuosity indices under a change in the frame center (macula, optic disc) of the eye fundus image. We illustrate the effects of such a change on 40 pairs of vessels evaluated with eight tortuosity indices. We show that the frame center change caused significant differences in the mean values of the vast majority of the tortuosity indices analyzed. The index defined as the ratio of the curvature to the arc length of a vessel segment proved to be the most robust in relation to a frame center change. Experimental results obtained from the analysis of clinical images are provided and discussed.

## 1. Introduction

The retinal blood vessels are directly and noninvasively observable through the crystalline medium of the eye. They have been shown to be one of the first structures directly affected by arterial hypertension and vascular dysregulation [1]. Their thickness, tortuosity and degree of deformation increase in response to cardiovascular overexertion caused by increased blood pressure [2]. The tortuosity of the retinal vessels can be used as a biomarker of several types of abnormalities, such as diabetic retinopathy [3], retinopathy of prematurity, glaucoma, macular degeneration, diabetes mellitus, hypertension, ischemic heart disease, some types of genetic disorders that affect hypertension and coronary artery disease [1,4,5], and other complications that can cause damage to the cardiovascular system (diabetes, coronary heart disease, etc.) [6]. Moreover, since the retinal vessels are an extension of the vessels of the brain, their appearance can be also related to the presence of cerebrovascular disease [5,7].

The retinal vascular network consists of a set of vessels, namely, arteries and veins, arranged in a double branching structure emerging from the optic disc, which is commonly the brightest spot in a retinography (eye fundus image). The first classifications of the general appearance of retinal vessels were based on subjective visual grading. It was not until 1979 that the first objective assessments of vessel tortuosity based on eye fundus photographs were performed [8]. Later, digital image processing applied to fundus images allowed the introduction of local indices for an enhanced objective measurement of retinal vessel tortuosity [9,10,11,12,13]. The usual local tortuosity indices are defined from a few geometrical features of the curve described by a vessel segment: curve length, distance between curve endpoints, total curvature, and total squared curvature [9,13].

The lack of a standardized protocol for image acquisition is still a limiting factor that hinders the assessment and its practical applicability to early diagnosis, progression monitoring, and treatment efficacy. Local tortuosity indices are calculated from retinographies with frames indistinctly centered on the macula (M) or on the optic disk (D) (see, for instance, the figures contained in [9,10,11,14,15]), with no mention of the effect that a frame center change might have on the values of the local tortuosity indices. In this work, we studied whether the center setting of the retinographies, either on the macula (M-retinography) or on the optic disc (D-retinography), has significant effects on the measurements of local tortuosity indices. To this end, we compared the values of eight widely known local tortuosity indices, as defined in [9,13], measured in forty vessels segmented from pairs of retinographies centered on the macula and the optic disc.

## 2. Materials and Methods

### 2.1. Local Tortuosity Indices

Local tortuosity indices measure the amount of twisting (ridges and valleys) of a vessel. They are commonly measured for a number of vessel segments presenting no bifurcation. These vessel segments can eventually contain bifurcation points, but not multiple branches.

Local tortuosity metrics of vessel segments are commonly defined from the ratios of some geometric parameters [9,13], such as chord length (D), arc length (L), total curvature (TK), and total squared curvature (TSK). L, TK, and TSK can be defined from a regular parametrization $C\left(t\right)$ of the curve C traced by the vessel segment. Regularity entails a continuous differentiability of $C\left(t\right)$ and the fulfillment of the condition on its derivative $C^{\prime }\left(t\right)\neq \vec{0}$, meaning that it presents no cusps or backtracks on itself. Let $C\left(t\right)=\left(x\left(t\right),y\left(t\right)\right)$, with $t_{0}\leq t\leq t_{1}$, be a regular curve describing the centerline of a vessel segment in terms of the 2D coordinate space $\left(x,y\right)$, the definitions of TK and TSK are based on the curvature $\kappa \left(t\right)$

(1) $$\kappa \left(t\right)=\frac{y^{\prime \prime }\left(t\right)\cdot x^{\prime }\left(t\right)-y^{\prime }\left(t\right)\cdot x^{\prime \prime }\left(t\right)}{{\left(x^{\prime }{\left(t\right)}^{2}+y^{\prime }{\left(t\right)}^{2}\right)}^{\frac{3}{2}}}.$$

**Figure 1:**
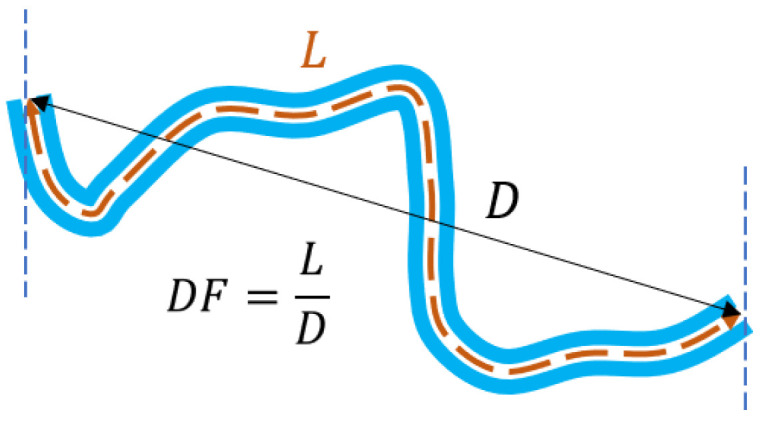
Illustration of the DF index as the ratio between the arc length (L ) and the chord length (D ), the latter being the Euclidean distance between the two endpoints of the vessel.

Table 1 shows the definitions of the four geometric parameters (D, L,TK, TSK). L, TK, and TSK are line integrals expressed in terms of the parametrization $C\left(t\right)$. Eight local tortuosity indices, beginning with the most widely used distance factor (DF), a variant thereof, T1, and the following T2…T7, have been defined from these geometric parameters [13], as shown in Table 2. Figure 1 illustrates further the DF index as “the relative length increase over a straight vessel” [13].

### 2.2. Participants and Equipment

**Figure 2:**
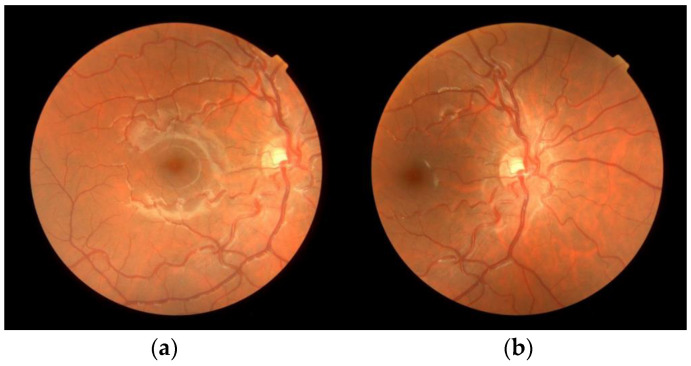
Original images. Retinographies of the pair (MR2/DR2) taken from subject 2′s right eye, with the frame centered on (**a**) macula (MR2); (**b**) optic disc (DR2).

To illustrate the problem, we analyzed 40 pairs of vessel segments extracted from the retinographies of two subjects {1, 2}. Two retinographies were acquired of each subject’s eye {left (L), right (R)}: one centered on the macula (M), and the other centered on the optic disc (D) (Figure 2). The set of eight images were labeled as reported in Table 3.

All images were acquired at the Ophthalmology Department at Mataró Hospital (Consorci Sanitari del Maresme, Barcelona, Spain) by a single experienced optometrist with a TRC-NW400 non-mydriatic retinal camera (Topcon Healthcare, Tokyo, Japan), in TIF format, RGB color, and a resolution of 768 × 806 pixels. Both subjects were selected among those regularly attending the service for the range of tortuosity observed in their retinas and the clarity of the vessel tree. We considered that their eye fundus images were useful for the purpose of our study. All the acquired images were anonymized by the hospital team before release. Figure 2 displays one of those pairs of images.

### 2.3. Region of Interest (ROI)

We cropped the original images for ROI selection. This way, the pixels were not altered—neither their RGB intensity values nor their geometrical features after extraction from each original retinography. Since we wanted to compare two different images of each individual vessel segment, it was essential to preserve the ROI content unaffected.

**Figure 3:**
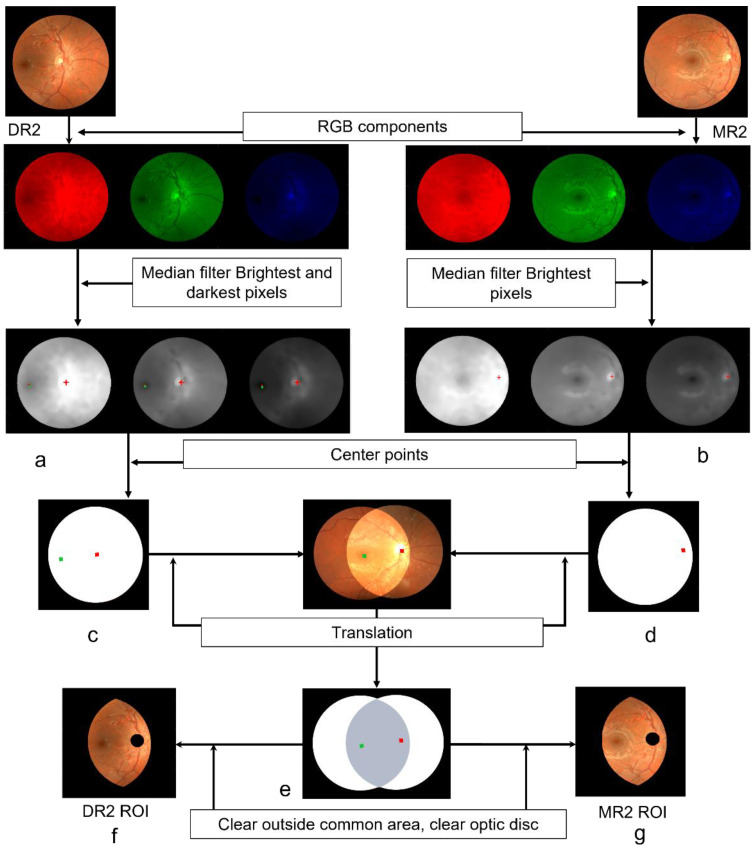
Determination of the ROI for DR2 and MR2. Brightest (red) points in all three RGB components of (**a**) DR2; (**b**) MR2. Darkest points are marked in green in (**a**). Optic disc midpoint (red) in (**c**) DR2; (**d**) MR2. Macula midpoint is marked in green in (**c**). (**e**) Intersection and common area (grey shaded) of DR2 and MR2. (**f**) ROI of DR2. (**g**) ROI of MR2. (See the text for more details).

Only vessel segments contained in both images of every M/D pair were interesting to explore the potential effect of changing the frame center on the tortuosity measurements. We developed an algorithm, based on binary masks, to roughly crop the common region of each M/D pair of images (Figure 3). Firstly, we cut each circular image of the retina by excluding the black corners of the square frame with an appropriate binary mask.

Next, we located the optic disc center in each retinography from its brightest point. To determine such a position, we smoothed the RGB components of each image with a median filter. A window of 21 × 21 pixels (about 3 times bigger than the thickest vessel diameter) was used to remove impulse noise and fine details from the image (Figure 3a,b). The brightest point of the image was calculated from the midpoint of the brightest pixels of the R, G, B components (red cross-shaped points in Figure 3a,b). In the case of having more than one pixel with maximum value in some component, the particular midpoint would be calculated for such a component prior to calculating the midpoint of the three R, G, B maxima for optic disc center location. This procedure yielded two optic disc center points: one for the M-retinography and the other for the D-retinography (red square-shaped points in Figure 3c,d).

An analogous procedure, but for the darkest point, allowed us to determine the macula center (green square-shaped point in Figure 3c) and hence, to calculate the distance between the optic disc and the macula center points in one of the images (arbitrarily chosen to be the D-retinography). We roughly estimated the radius of the optic disk as one fifth of such a distance [16].

We translated either image to make their optic disk center points coincide and clear outside the overlapping area (gray-shaded region in Figure 3e). Additionally, we used the estimated optic disc radius to clear it with a circular mask in both images. The resulting ROIs (DR2-ROI and MR2-ROI in Figure 3f and Figure 3g, respectively) provided us with two image versions of each vessel segment whose tortuosity was to be evaluated.

### 2.4. Vessel Segmentation and Parametrization

**Figure 4:**
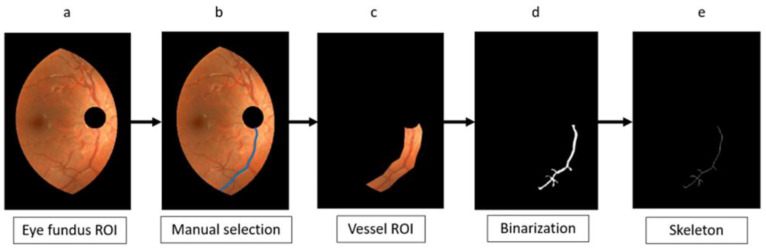
Flow chart for vessel-ROI segmentation, binarization, and skeletonization: from the eye fundus ROI (**a**), a vessel is manually selected (**b**). The vessel ROI is segmented (**c**), binarized (**d**), and skeletonized (**e**).

Figure 4 illustrates the segmentation process starting from DR2-ROI (Figure 4a). We developed a Matlab (Mathworks, Natick, MA, USA) tool to assist in the manual selection of the 40 vessel segments included in this study. The Matlab tool allowed the user to roughly draw a free-hand line on a vessel path (blue line in Figure 4b), between two formerly selected endpoints. The purpose of this line was to create a specific ROI for the vessel segment by padding the line with a surrounding area of 20 pixels (let us recall that it means about three times bigger than the thickest vessel diameter), in both the X and the Y directions (Figure 4c). The endpoints’ coordinates were recorded as tentative values and later used to refine the final endpoint positions of the segment. To make it more accurate, we considered endpoints clearly identifiable in both retinographies, so they were limited to bifurcation points, crossing points, the optic disk edge, the general ROI edge, or branch endpoints. This condition for the endpoints aimed to reduce subjective inaccuracies in the double vessel-ROI selection of each segment.

The part of the vessel tree contained in that vessel-ROI was binarized (Figure 4d) following the method described in [17]. This is an unsupervised binarization method that overcomes the common problem of non-uniform illumination of eye fundus images. The method follows with an iterative algorithm that starts with a seed and adds, at each iteration, a new vessel segment connected to the previously segmented part. The result preserves the connectivity as a distinct feature of the retinal vessel tree. For the current work, the described method [17] provided the segmentation of the part of the tree contained in the vessel-ROI and, hence, the vessel segment of interest. To smooth small irregularities, the result was improved with a morphological closing operation (Figure 4d). A circular structural element with a radius of 7 pixels (the thickest vessel width) was used for that closing. Next, we skeletonized the segmented part of the vessel tree (Figure 4e).

**Figure 5:**
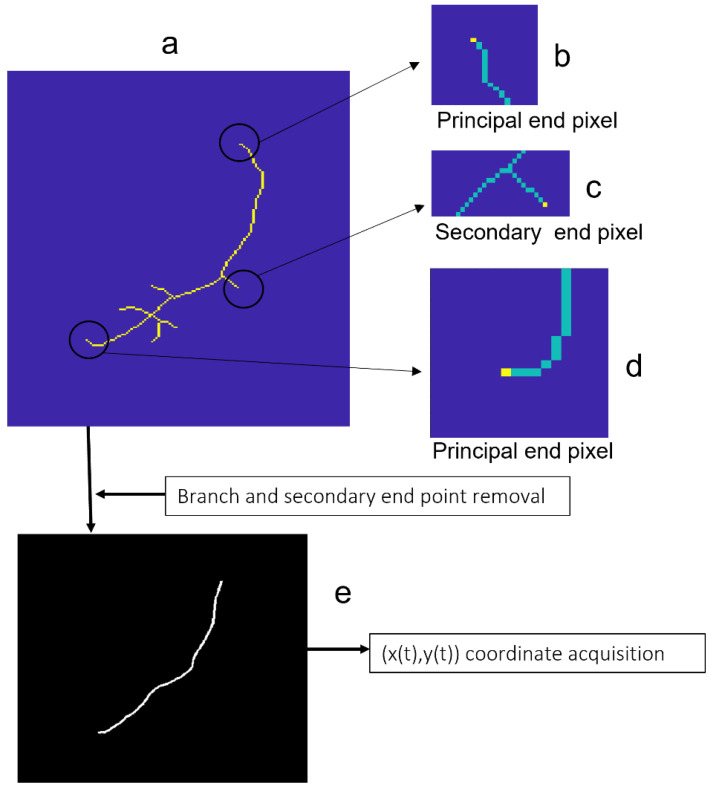
Vessel segment refinement, coordinate acquisition, and parametrization. (**a**) Skeletonized element; Endpoints: principal (**b**,**d**), secondary (**c**); (**e**) 1-pixel wide curve of connected pixels running along the central line of the vessel segment for coordinate acquisition.

Endpoints, bifurcation points, and crossing points were recognized in the skeletonized element (Figure 5a) using the following criteria: pixels with only one neighbor pixel were labeled as endpoints (in yellow in Figure 5b–d), whereas pixels with three or more neighbor pixels were labeled as tree-branching pixels (either bifurcation or crossing). This set of labeled pixels should contain the two principal endpoints of the vessel segment, which could differ slightly from the previously recorded as tentative endpoints. From all the pixels labelled as end, bifurcation, and crossing point pixels in the skeletonized vessel, we eventually identified the two principal endpoints of the vessel segment as those being the closest to the tentative ones. Needle branches were removed by an iterative procedure: secondary endpoints, that is, endpoints other than the principal, were removed from the skeleton; next, new tree secondary endpoints of the remaining skeleton were found and further removed. The procedure was repeated until no endpoints other than the principal ones remained. As a result, we obtained a curve, 1-pixel wide of connected pixels, running along the central line of the selected vessel segment (Figure 5e).

**Figure 6:**
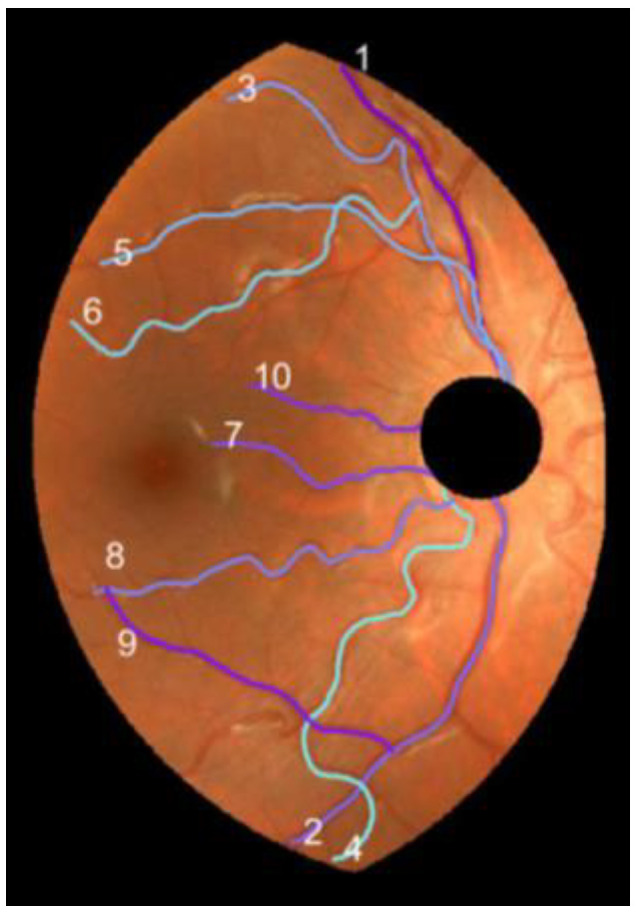
Vessel segments (1, ..., 10) selected in DR2.

We proceeded to the parametrization of the resulting line of the vessel segment. We built a string with their pixel coordinates. The first element of the string was arbitrarily assigned to the principal endpoint closest to the (0, 0) pixel of the original image. It was followed by the next neighbor pixel of the line and so on. Note that the parameter runs from t=1 to the total number of pixels of the vessel segment. A final smoothing operation with a 3-term moving average completed the parametrization of the vessel segment. Figure 6 displays the 10 vessels selected from the retinography DR2.

### 2.5. Vessel Curvature

Besides the chord (D) and arc (L) lengths (Table 1 and Figure 1), the definition of other tortuosity indices (Table 2) involves total curvature (TK) and total squared curvature (TSK). Curvature is defined from the first and second derivatives of the curve coordinates, and their direct calculation as differences between consecutive terms usually displays a noisy behavior. Smoothing is frequently used to handle this issue, but it can alter the geometry of the curve when is applied excessively.

We estimated the first and second derivatives following a robust method against small perturbations of the curve, described in [18]. The method uses the second-order Taylor expansion of the coordinate functions and the weighted least squares method. For each point of a string $\left(x\left(m\right),y\left(m\right)\right)$ and for p=±4,±8,±12,±16,±20, we obtained two sets of 10 equations from the second-order Taylor expansion (the number of equations decreased down to 5 for points close to the principal endpoints):

(2) $$x\left(m+p\right)=x\left(m\right)+x’\left(m\right)\cdot p+\frac{1}{2}\;x’’\left(m\right)\cdot p^{2}+\varepsilon_{x}\left(m,p\right),\;p=\pm 4,\pm 8,\pm 12,\pm 16,\pm 20,$$

(3) $$y\left(m+p\right)=y\left(m\right)+y’\left(m\right)\cdot p+\frac{1}{2}\;y’’\left(m\right)\cdot p^{2}+\varepsilon_{y}\left(m,p\right),\;p=\pm 4,\pm 8,\pm 12,\pm 16,\pm 20.$$

We set p values taking into account that 4 pixels corresponded roughly to half the width of the thickest vessel. The terms $\varepsilon_{x}\left(m,p\right)\;y\;\varepsilon_{y}\left(m,p\right)\;$ represent higher order contributions in Equations (2) and (3). Both systems of equations were solved using weighted least squares with weights equal to $\frac{1}{\sqrt{p}}$, which assigned lower weight to equations corresponding to farther neighbor pixels. Equation (2) was solved for $x’\left(m\right)$ and $x’’\left(m\right),$ and Equation (3) for $y’\left(m\right)$ and $y’’\left(m\right)$. The sequences $x’\left(m\right)$, $x’’\left(m\right)$, $y’\left(m\right)$, and $y’’\left(m\right)$ were finally smoothed using a 3-pixel moving average operator. The resulting derivatives were used to calculate the curvature $\kappa \left(t\right)$ (Equation (1)).

## 3. Results

**Figure 7:**
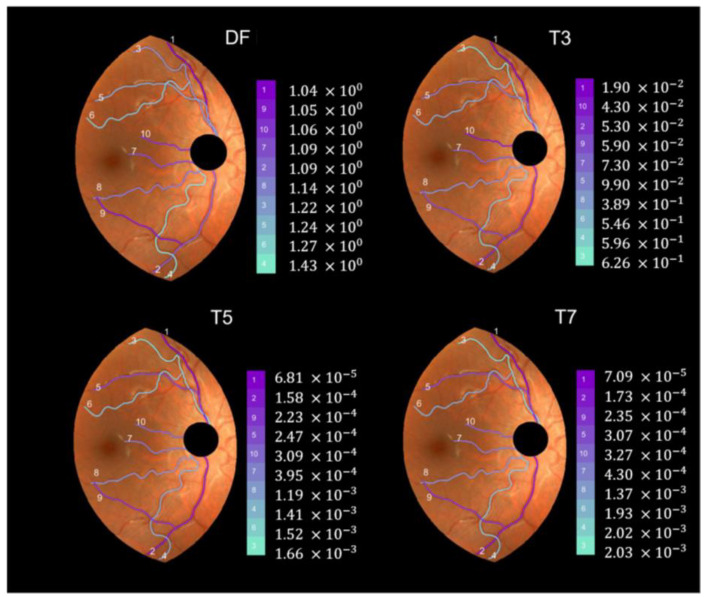
Individual tortuosity values (DF, T3, T5, and T7 indices) for the 10 selected vessels in DR2-ROI (D-retinography of subject 2′s right eye).

We applied the method described in the previous section to the set of 40 pairs of vessel segments selected from the D- and M- retinographies of both (R, L) eyes of subjects 1 and 2. To illustrate the results, Figure 7 shows the individual values of the indexes DF, T3, T5, and T7 for the ten vessel segments selected from DR2. They appear colored according to the grade of tortuosity (tortuosity increases from dark magenta to light blue). For the vessels in Figure 7, the four indices showed different values. Three of them (T3, T5, and T7) coincided in pointing vessel 3 as the most tortuous, while the DF index indicated vessel 4. The T5 and T7 indices very similar (only vessels 4 and 6 appeared swapped in second and third positions from the highest tortuosity). All four indices agreed in marking vessel 1 as the least tortuous of the group.

**Figure 8:**
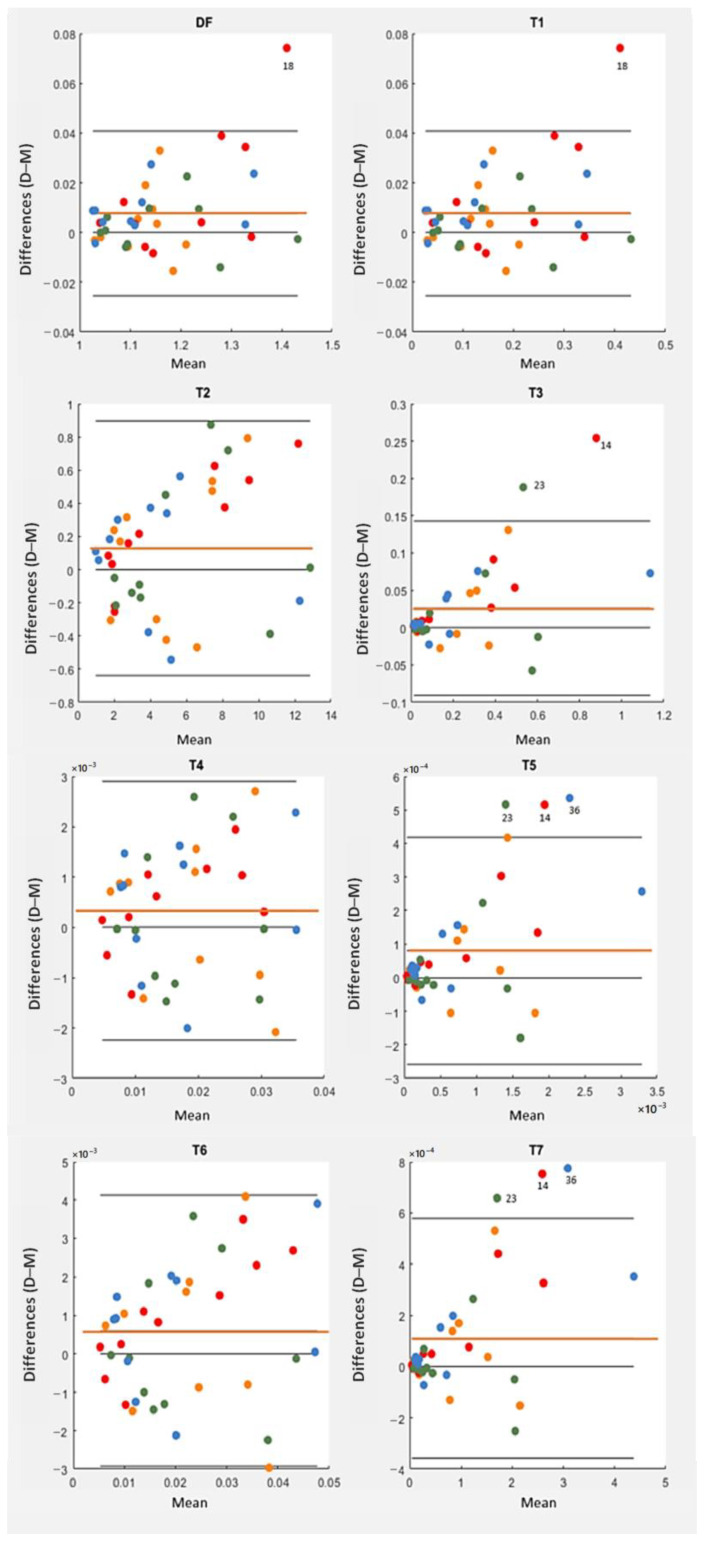
Bland–Altman plots for each tortuosity index (DF, T1 … T7). Differences between the values obtained in D-retinographies and the values obtained in M-retinographies are represented on the vertical axis. In each plot, the orange line represents the mean value of the individual differences. Points of the same color correspond to the same eye. Dot colors represent eye and subject, being MR1/DR1 (orange), ML1/DL1 (red), MR2/DR2 (green), ML2/DL2 (blue). Point 14 represents vessel 4 in ML1/DL1, point 18 vessel 8 in ML1/DL1, point 23 vessel 3 in MR2/DR2, and point 36 vessel 6 in ML2/DL2.

Table 4 contains the mean and standard deviation values of the tortuosity indices computed for the set of vessel segments in either D- or M- retinography. From the table, it stands out that the indices were expressed in very different scales, even orders of magnitude apart; therefore, their direct comparison is not meaningful. For each index, their statistical values seemed to be quite similar for the D- and M- frame centers, but a detailed analysis of the individual differences using Bland–Altman plots (Figure 8) and paired t-tests (Table 5) revealed significant differences for most of them.

We also analyzed any possible effect of the specific eye under examination on the individual differences of the tortuosity indices. All the points corresponding to a given eye share the same color in Figure 8. For each tortuosity index, a one-factor ANOVA showed no significant differences between the eyes. One-factor ANOVA tests compared the four means of the tortuosity differences between pairs of vessels, a mean value for each eye. These tests are suited to detect heterogeneity across eyes.

For each tortuosity index, the Bland–Altman plot showed systematic differences caused by a D-to-M frame center change. The X-axis accounts for the amount of tortuosity (mean value), and the Y-axis for the individual differences. In each plot, the central line represents the mean value of the individual differences (second column in Table 5) and denotes the systematic difference (bias) caused by the frame center change: all the tortuosity indices had a positive bias, that is, reached higher values, on average, when they were evaluated in D- rather than in M-retinographies. The upper and lower lines are the limits of concordance (mean of the differences ± 1.96 * standard deviation of the differences, in the fourth and fifth columns of Table 5). All systematic differences were statistically significant (i.e., different from 0, with *p*-values < 0.05 in Table 5), except for T4. In other words, the mean tortuosity values resulting from either M- or D-retinographies were significantly different for all tortuosity indices, excluding T4. The higher *p*-values (0.040, 0.112, 0.038) correspond to tortuosity indices based not on $\kappa^{2}$, but on linear κ (T2, T4, and T6).

**Figure 9:**
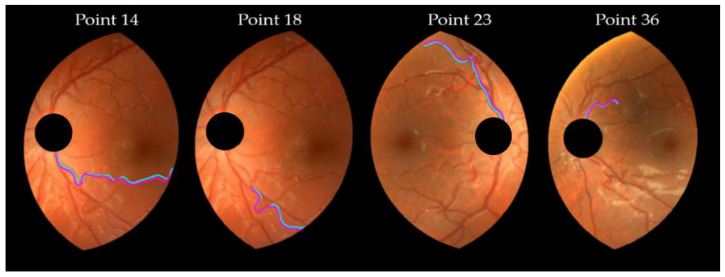
Pairs of vessel segments. Point 14 is vessel 4 in ML1/DL1, point 18 is vessel 8 in ML1/DL1, point 23 is vessel 3 in MR2/DR2 (see Figure 6), and point 36 is vessel 6 in ML2/DL2). They fall off the limits of concordance in some Bland–Altman plots (Figure 8). Magenta (cyan) lines correspond to M-retinography (D-retinography).

From the definition of the concordance limits in the Bland–Alman plot, about 5% of the differences caused by the frame center change should lie outside the limits of agreement presented in Table 5. Three points fell beyond the limits in the T5 and T7 plots, very close to the two (5%) expected points. The DF and T1 plots are identical, as a consequence of the T1 definition (T1=DF−1, see Table 2). The plots showed higher differences for higher tortuosity values, although this behavior was mild with T4. Therefore, the variability increased with the magnitude of the tortuosity measure. The points corresponding to 18, 14, 23, and 36 pairs of vessels, were fairly beyond the limits of concordance in some plots of Figure 8. They corresponded to moderate to high tortuosity values. The corresponding pairs of vessels appear redrawn in magenta (vessel from M-retinography) and cyan (vessel from D-retinography) in Figure 9. The cyan lines seem to have more hairpin turns than the magenta ones; therefore, the cyan lines computed higher curvature values. This was confirmed by the geometrical features *TK* and *TSK* of those vessels, listed in Table 6. Moreover, in D-retinographies, their lengths *L* (except for the 23) appeared to be greater, while the chords *D* appeared to be shorter. These two facts, longer *L* and shorter *D* chord, led to more twisted lines.

Since the values reached by the set of indices in Table 4 were not straightforwardly comparable, we analyzed the consistence of the indices through the Spearman rank correlation coefficient (ρ). For consistence, it is meant that a vessel characterized by a high value of a specific tortuosity index would also present a high value of the other tortuosity indices. In other words, the list of vessels ordered by their tortuosity using a specific index should be equal or very similar to the list obtained considering other indices. Table 7 contains the Spearman rank correlation coefficient and the Pearson correlation coefficient (r) for pairs of tortuosity indices. Let us recall that r assesses the linear relationship between two tortuosity indices. As expected from their definition, an exact coincidence of tortuosity rank orders was found for DF and T1 (ρ=1, r=1), closely followed by T5 and T7 (ρ=0.998, r=0.946). The lowest values of both the Spearman and the Pearson correlation coefficients were found for T4 and DF or T1 (ρ=0.775, r=0.758).

**Figure 10:**
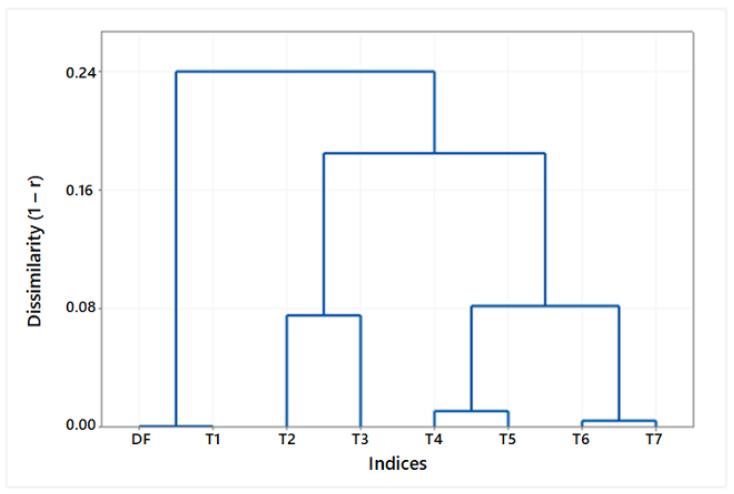
Dendrogram showing the dissimilarity (1−r) among tortuosity indices according to Pearson’s correlation coefficient and the resulting clusters.

Finally, a dendrogram (Figure 10) summarizes the similarities among the set of tortuosity indices. The tree diagram displays the groups arising from an iterative clustering of the tortuosity indices. The dissimilarity level (1−r) according to Pearson’s correlation coefficient is represented on the vertical axis, and the indices are listed on the horizontal axis. The lowest correlation was found between the cluster of indices based on the vessel length (DF, T1) and the cluster of indices based on the vessel curvature (T2 … T7). Within this second cluster, the biggest dissimilarity was observed between the subgroup of total curvature measurements (T2, T3) and the subgroup of relative curvature measurements (T4 … T7), concerning either *TK* (T4, T6) or *TSK* (T5, T7).

## 4. Discussion and Conclusions

The tortuosity of a retinal vessel tree can be analyzed on retinographies with a frame center either on the macula (M) or on the optic disk (D). No recommendation or standard protocol has been found to use one or another for image acquisition. We have analyzed the effect of a frame center change on the tortuosity values measured through eight local tortuosity indices already introduced in the field and widely used in related literature [13].

To illustrate the issue with examples, we selected 40 vessel segments from the clinical fundus images of two subjects’ eyes, ten segments per eye. Two separate retinographies, M- and D-centered, of each eye, provided a pair of parametric descriptions for the centerline of each vessel segment. Vessel segments were selected to have easily identifiable endpoints in both retinographies and also to exemplify a varied grade of tortuosity.

Our results showed that a frame center change affected significantly the tortuosity measures of almost all indices of the set. The tortuosity indices reached higher values when they were evaluated through a D-retinography than through an M-retinography. The differences were statistically significant for all the indices tested, except for the index T4, which is based on the total curvature (*TK*) divided by the arc length (*L*).

The Bland–Altman plots also showed unwanted behaviors of the individual differences. For all the indices, the standard deviation tended to increase across the tortuosity magnitude value, producing inverted funnel-shaped plots. The effect was mild with T4. However, the inverted funnel was clear with the DF (and its variant T1) indices, as well as with the indices based on the squared curvature (*TSK*) (T3, T5, and T7). For them, we also analyzed the cases of extreme differences, meaning those points beyond the tortuosity limits of concordance in the Bland–Altman plots. They corresponded to vessels with higher total curvatures (for most of them, longer arc lengths too) and shorter chord lengths in the D-retinographies than in the M-retinographies.

The tortuosity index T4 was the most robust when performing a frame center change (M- to D-) among the eight indices analyzed. Based on the Spearman correlation coefficient (ρ), the rank order obtained with T4 was very similar to those derived from the other curvature-based indices T3 (0.921), T5 (0.983), T6 (0.995), and T7 (0.982); however, the rank ordered obtained with T4 showed the maximum difference with respect to that derived from DF (and T1) (0.775).

When analyzing retinographies with either D or M frame center, the tortuosity index T4 appeared to compensate better for the tiny effects of the perspective change on the parametric description of the vessels and, hence, on their local tortuosity measures. The DF index (and T1), not based on the vessel curvature, showed significant differences when evaluated in either a D- or an M-retinography. This fact needs to be taken into account, since DF and its variant T1 are conceptually simple and the most widely used tortuosity measures [9,13,19]. Moreover, its use has been objected as it may underestimate vessel tortuosity, as reported by Kalitzeos et al. [13] and formerly by Aslam et al. [20]. The rest of the tortuosity indices (T2, T3, T5, ... T7), though based on the curvature (*TK* or *TSK*) and, some of them, also divided by either the arc (T5) or the chord (T6, T7) length (Table 1 and Table 2), do not capture the same representation of a vessel tortuosity with the change of the frame center. In the dendrogram (Figure 10), DF and T1 appear as redundant. T4 and T6, on the one hand, and T5 and T7, on the other hand, are very similar, as they only differ in the sort of length magnitude used in denominator (arc or chord length). On the following level, the curvature-based indices (T2, T4, T6) are dissimilar to the square curvature-based indices (T3, T5, and T7), and yet, those absolute curvature-based indices (T2, T3) in a higher level of dissimilarity from the indices with curvature relative to a length magnitude (T4, T5, T6, T7). In the highest level of dissimilarity, we find the DF (T1) index separated from the rest of curvature-based indices (T2 … T7).

This work has two obvious limitations. Firstly, although we found significant differences in most of the indices used to evaluate retinal vessel tortuosity when changing the fundus frame center (macula, optic disc), we ignored the impact such a difference may have in the clinical practice. We think this fact deserves to be investigated. Secondly, we analyzed just 40 pairs of vessel segments to provide an example that may illustrate the issue and motivate further research. The investigation should be extended to cover a variety of cases, in particular, those with clinical interest. Finally, and most importantly, these results should lead to establish standard protocols for eye fundus acquisition relative to the evaluation of retinal vessel tortuosity.

## Figures and Tables

**Table 1:** Geometric parameters of a curve parametrized by $C\left(t\right)$.

Chord length	$D=\sqrt{{\left(x\left(t_{1}\right)-x\left(t_{0}\right)\right)}^{2}+{\left(y\left(t_{1}\right)-y\left(t_{0}\right)\right)}^{2}}$
Arc length	$L=\int_{C}^{\;}1ds=\int_{t_{0}}^{t_{1}}1\cdot \sqrt{{\left(x’\left(t\right)\right)}^{2}+{\left(y’\left(t\right)\right)}^{2}}\;dt$
Total curvature	$TK=\int_{C}^{\;}\left|\kappa \right|ds=\int_{t_{0}}^{t_{1}}\left|\kappa \left(t\right)\right|\cdot \sqrt{{\left(x’\left(t\right)\right)}^{2}+{\left(y’\left(t\right)\right)}^{2}}\;dt$
Total squared curvature	$TSK=\int_{C}^{\;}\kappa^{2}ds=\int_{t_{0}}^{t_{1}}\kappa^{2}\left(t\right)\cdot \sqrt{{\left(x’\left(t\right)\right)}^{2}+{\left(y’\left(t\right)\right)}^{2}}\;dt$

**Table 2:** Definition of eight common local tortuosity indices [13].

$DF=\frac{L}{D}$	$T1=\frac{L}{D}-1$	T2=TK	T3=TSK
$T4=\frac{TK}{L}$	$T5=\frac{TSK}{L}$	$T6=\frac{TK}{D}$	$T7=\frac{TSK}{D}$

**Table 3:** Retinography labels according to subject, eye, and frame center.

	Right Eye		Left Eye	
Frame Center	Macula	Optic Disc	Macula	Optic Disc
Subject 1	MR1	DR1	ML1	DL1
Subject 2	MR2	DR2	ML2	DL2

**Table 4:** Mean and standard deviation for the 40 pairs of selected vessel segments of each index and frame center (M, macula and D, optic disc).

	Mean		Standard Deviation	
Index	M	D	M	D
DF	1.1543	1.1621	0.1101	0.1169
T1	0.1543	0.1621	0.1101	0.1169
T2	4.935	5.063	3.307	3.427
T3	0.2085	0.2344	0.2412	0.2756
T4	0.01709	0.01742	0.00915	0.00927
T5	0.000694	0.000774	0.000718	0.000811
T6	0.02046	0.02105	0.01251	0.01292
T7	0.000860	0.000970	0.000952	0.001087

**Table 5:** Mean and standard deviation of individual differences. Limits of agreement and *p*-value of the paired *t*-test for the tortuosity indices. *p*-value > 0.05 appeared boldfaced.

Tortuosity Indices	Mean Difference	Standard Deviation	Limits of Agreement		Limits of Agreement
			Lower	Higher	
DF	0.00768	0.016570	−0.02480	0.04016	0.006
T1	0.00768	0.016570	−0.02480	0.04016	0.006
T2	0.12910	0.384400	−0.62430	0.88252	0.040
T3	0.02591	0.058390	−0.08850	0.14035	0.008
T4	0.00033	0.001285	−0.00220	0.00285	**0.112**
T5	0.00008	0.000169	−0.00025	0.00041	0.005
T6	0.00060	0.001764	−0.00285	0.00405	0.038
T7	0.00011	0.000234	−0.00035	0.00057	0.005

**Table 6:** Geometrical features of the vessels in Figure 9.

Vessel	Frame Center	*D*	*L*	*TK*	*TSK*
14	DR2	339.86	457.30	12.57	1.007
	MR2	340.51	446.45	11.81	0.752
18	DR2	187.58	271.50	8.31	0.520
	MR2	190.69	261.84	7.93	0.466
23	DR2	308.30	377.31	7.79	0.627
	MR2	318.91	383.10	6.92	0.439
36	DR2	102.46	139.02	5.09	0.356
	MR2	103.79	138.37	4.75	0.280

**Table 7:** Spearman rank correlation (ρ ) and Pearson correlation (r ) coefficients computed for pairs of the tortuosity indices.

		DF	T1	T2	T3	T4	T5	T6
T1	ρ r	1.000 1.000						
T2	ρ r	0.830 0.800	0.830 0.800					
T3	ρ r	0.827 0.758	0.827 0.758	0.975 0.924				
T4	ρ r	0.775 0.758	0.775 0.758	0.869 0.826	0.921 0.817			
T5	ρ r	0.790 0.760	0.790 0.760	0.890 0.815	0.954 0.919	0.983 0.932		
T6	ρ r	0.814 0.834	0.814 0.834	0.890 0.846	0.938 0.843	0.995 0.990	0.985 0.943	
T7	ρ r	0.808 0.791	0.808 0.791	0.896 0.814	0.957 0.922	0.982 0.918	0.998 0.946	0.987 0.941

## Data Availability

The authors declare their will to provide further data supporting the reported results upon reasonable request.
